# Quantitative Assessment of Desertification Using Landsat Data on a Regional Scale – A Case Study in the Ordos Plateau, China

**DOI:** 10.3390/s90301738

**Published:** 2009-03-12

**Authors:** Duanyang Xu, Xiangwu Kang, Dongsheng Qiu, Dafang Zhuang, Jianjun Pan

**Affiliations:** 1 Collage of Resources and Environmental Sciences, Nanjing Agricultural University, Nanjing 210095, Jiangsu Province, P.R. China; E-Mails: duanyang_xu@126.com (D.X.); jpan@njau.edu.cn (J.P.); 2 Institute of Scientific and Technical Information of China, Beijing 100038, P.R. China; E-Mail: kangxw@istic.ac.cn (X.K.); 3 State Key Lab of Resources and Environmental Information System, Institute of Geographical Sciences and Natural Resources Research, Chinese Academy of Sciences, Beijing 100101, P.R. China; E-Mail: qiuds@igsnrr.ac.cn (D.Q.)

**Keywords:** Desertification, quantitative assessment, Landsat, Ordos Plateau

## Abstract

Desertification is a serious threat to the ecological environment and social economy in our world and there is a pressing need to develop a reasonable and reproducible method to assess it at different scales. In this paper, the Ordos Plateau in China was selected as the research region and a quantitative method for desertification assessment was developed by using Landsat MSS and TM/ETM+ data on a regional scale. In this method, NDVI, MSDI and land surface albedo were selected as assessment indicators of desertification to represent land surface conditions from vegetation biomass, landscape pattern and micrometeorology. Based on considering the effects of vegetation type and time of images acquired on assessment indictors, assessing rule sets were built and a decision tree approach was used to assess desertification of Ordos Plateau in 1980, 1990 and 2000. The average overall accuracy of three periods was higher than 90%. The results showed that although some local places of Ordos Plateau experienced an expanding trend of desertification, the trend of desertification of Ordos Plateau was an overall decrease in from 1980 to 2000. By analyzing the causes of desertification processes, it was found that climate change could benefit for the reversion of desertification from 1980 to 1990 at a regional scale and human activities might explain the expansion of desertification in this period; however human conservation activities were the main driving factor that induced the reversion of desertification from 1990 to 2000.

## 1. Introduction

Desertification is recognized as one of the most serious social-economic-environmental issues in arid, semi-arid and dry sub-humid areas, and in more than one hundred countries, about one billion of the six billion world population are affected by desertification [1,2]. Since the United Nations Conference on Desertification held in 1977, the definition and assessing methodology for desertification have been controversial. Although the definition of desertification put forward by United Nations Convention to Combat Desertification [3] in 1994, that “desertification is land degradation in arid, semiarid and dry sub-humid areas resulting from various factors, including climatic variations and human activities” has been widely accepted, there is no consensus concerning assessment systems and methodology on the proper way to assess it and this may result in desertification being measured by different researchers using different assessment methods that cannot be compared for the same region [4]. Meanwhile, there is a potential problem that qualitative indicators and assessment methods may produce human-induced errors when assessed by different people, which can also reduce accuracy for desertification measurement. Therefore, there is a pressing need to develop a quantitative methodology, especially from a remote sensing point of view, to accurately measure desertification on both regional and global scales.

Climatic variations including rainfall, temperature, sunlight, wind etc. and human activities like intensive land use, overgrazing, over-cutting etc. can lead to changes in land surface conditions during desertification processes [5–8]. As a consequence of climatic variations and human activities that affect desertification, vegetation conditions in arid, semiarid and dry sub-humid environment have greatly changed, and these changes not only include the reduction of vegetation cover, density and biomass, but also are characterized by structural configurations of vegetation types and landscape patterns [5,9,10]. Vegetation dynamics can also induce the change of soil properties including physical and chemical properties of soil [11,12], and micrometeorological conditions of land surface like temperature, albedo etc. [13], which usually have been used to characterize desertification. These changes of land surface conditions make the spectral characteristics of desertification land vary greatly to different degrees, which can be captured by satellite sensors and this may be fundamental for quantitatively assessing desertification by means of the indices derived from satellite images [14]. Because the accuracy of soil properties acquired from satellite images is very variable [15,16], much previous research has focused on constructing image-based indices to retrieve vegetation and micrometeorological conditions of land surface to monitor desertification at different scales [17–19].

Image-based vegetation indices such as Normalized Difference Vegetation Index (NDVI), Soil Adjusted Vegetation Index (SAVI), and Modified SAVI (MSAVI) are virtually always used to assess vegetation conditions in desertification monitoring [18,20]. Meanwhile, some indices that can reflect vegetation patterns and spatial heterogeneity, such as the landscape connectivity index [21], Moving Standard Deviation Index (MSDI) [22,23] etc. are adopted for desertification assessment. There are classical methods for retrieving the micrometeorological conditions of land surfaces such as land surface temperature (LST) and albedo from satellite image by using thermal infrared band or other band combinations to accurately get micrometeorological parameters of land surfaces [20,24]. Based on these indices retrieved from images, various assessment models such as decision tree classification [20], unsupervised classification [25], Radial Basis Function Network (RBFN) [26] etc. were used to determine desertification status. However, many researchers have focused on assessment indicators or methods themselves, but little research has been conducted on the effect of environment heterogeneity and seasonal variation on the values of assessment indicators. Liu divided North China into four sub-regions according to climate variation and built different indices system for each region to assess desertification [20], but to the authors’ knowledge few researchers have made similar attempts on a regional scale.

Landsat series data can provide long-term, high quality multi-band images for environmental monitoring and assessment at regional, national and global scales [27,28]. In this paper, we selected the Ordos Plateau in China as the research region and attempted to develop a quantitative method for desertification assessment by developing indicators from Landsat data that consider the effect of environment heterogeneity and seasonal variation on values determined using the indicator system.

## 2. Study Area

**Figure 1.**
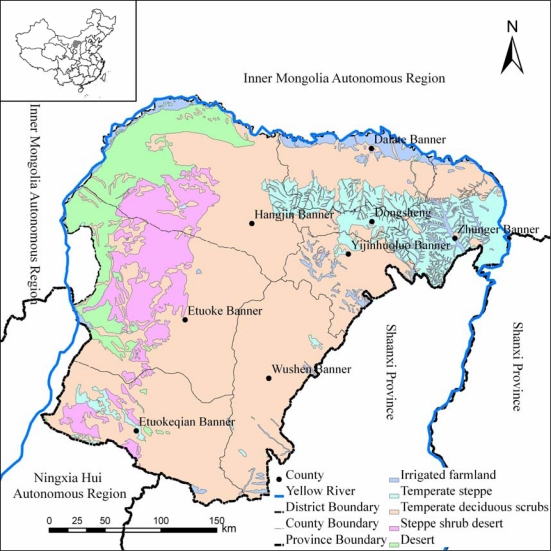
General location of study area: Ordos Plateau.

The Ordos Plateau is located in the southwest of the Inner Mongolia Autonomous Region of China, ranging from 37°41′∼40°51′ N and 106°42′∼111°31′ E (Figure 1). Ordos Plateau (hereinafter shortened to “Ordos”) has an area of approximately 86,752 km^2^ and population about 1,395,000. It contains an ecotone with air circulation changing from temperate continental climate, high-pressure ridge (Mongolia-Siberian) in the northwest to the influence of monsoonal trough in the southeast. This air circulation makes Ordos have the climate with an annual sunshine duration of 2,716–3,194 hours; an average annual temperature of 5.3–8.7°C, and an annual evaporation of 2,000–3,000mm. The average annual rainfall of Ordos is 170–450 mm, which varies from west to east with most of the annual rainfall falling from July to September. The climate transition in Ordos make its vegetation diverse and mainly includes five vegetation or landscape types: irrigated farmland along Yellow River in the north, where dominated vegetation type is crop such as corn and wheat; temperate steppe in the east, where the plant community coverage can reach 40–50% and the dominant grass species are *Stipa bungeana, Aster altaicus and S. breviflora;* temperate deciduous scrubs in the southeast, where covers a majority of Mu Us sandy land and the dominant scrub species are *Artemisia giraldii* and *Caragana korshinskii*; steppe shrub desert in the southwest, where shrub and semi-shrub species are usually lower and dominant specie is *Caragana tibetica*; desert in the northwest, where covered by drift dune and the dominant species are *Agriophyllum squarrosum*, *Corispermum spp.*, *salsola spp.*. Long-term intensive land use and dry climate condition make desertification become the most serious environmental problem in Ordos, which has Mu Us sandy land (in the southeast) and Qubqi desert (in the northwest) covering approximately 43,000 km^2^, and accounting for 48% of the whole region [29,30].

## 3. Materials and Methodology

### 3.1. Data Source

Landsat data including seven scenes (September 1st, 1979, path/row 137/32; August 19, 1978, path/row 137/33; August 16, 1980, path/row 138/32; October 9, 1980, path/row 138/33; August 20, 1978, path/row 138/34; September 22, 1980, path/row 139/32; October 9, 1979, path/row 139/33) of Landsat MSS (Multi Spectral Scanner, MSS) in 1980 (here 1980 means the period before and after 1980 and the same meaning as that in 1990 and 2000), seven scenes (August 13, 1990, path/row 127/32; September 11, 1989, path/row 127/33; August 7, 1991, path/row 128/32; September 15, 1990, path/row 128/33; August 23, 1991, path/row 128/34; August 24, 1989, path/row 129/32; August 30, 1991, path/row 129/33) of Landsat TM (Thematic Mapper, TM) in 1990 and seven scenes (June 13, 2000, path/row 127/32; August 6, 2000, path/row 127/33; November 11, 2000, path/row 128/32; November 11, 2000, path/row 128/33; September 22, 1999, path/row 128/34; June 11, 2000, path/row 129/32; August 12, 1999, path/row 129/33) of Landsat ETM (Enhanced Thematic Mapper plus, ETM+) in 2000 covering all of Ordos were used for monitoring desertification from 1980 to 2000. All spectral data were collected in summer and autumn, mainly from June to November. Radiometric calibration, geometric correction, and cloud removal were carried out for all of these images, which were all geo-referenced to the WGS_1984_UTM Projected Coordinate System with a geometric precision of 0.5 pixel. A 1:1,000,000 vegetation map of Ordos was acquired from the 1:1,000,000 vegetation map of China, which was used for vegetation and landscape classification in Ordos. Meteorological data including monthly averaged temperature, rainfall, etc. of thirty-four meteorological stations in and around Ordos were collected from China Central Meteorological Bureau. Social-economic census data of Ordos were collected from local statistical yearbooks.

### 3.2. Indicator Selection and Acquisition

The changes of land surface conditions induced by desertification are mainly characterized by the change of vegetation biomass or cover, landscape pattern and micrometeorological conditions. Therefore, the indicators that reflect these changes can be selected to assess desertification. Considering the strong correlation among indicators in assessing one aspect of land surface condition, such as among NDVI, SAVI and vegetation cover in assessing vegetation biomass [31], we only selected one indicator to represent one aspect of land surface conditions. In this study, NDVI, MSDI and land surface albedo were used to characterize vegetation biomass, landscape pattern and micrometeorological conditions of land surface respectively.

In this study, all of the three indicators were acquired through analysis of Landsat images for desertification assessment. To quantify vegetation biomass for desertification, NDVI was selected and calculated as the ratio of the difference between near infrared (*NIR*) and red (*RED*) bands to the sum of them (Formula 1):

(1) $$\mathrm{NDVI}=\frac{\mathrm{NIR}-\mathrm{RED}}{\mathrm{NIR}+\mathrm{RED}}$$

Desertification is characterized not only by the change of vegetation biomass, but also by the change of landscape pattern or heterogeneity. In this study, landscape pattern or heterogeneity in desertification was assessed by using MSDI, which had been proven to be a robust indicator to assess landscape heterogeneity for land degradation. The MSDI was calculated by passing a 3×3 filter across red wavelengths band (MSS band2 and TM/ETM+ band3) and calculating the standard deviation for every nine-pixel window. Then the standard deviation was placed onto a new map at the same location as the central pixel of each nine-pixel window (Formula 2) [22,23]. Where *N* represent pixel number of filter, and here *N* =9; *DN_i_* is the digital number of pixel *i* in each nine-pixel window and $\bar{\mathrm{DN}}$ is the average digital number value of each nine-pixel window:

(2) $$\mathrm{MSDI}=\sqrt{\frac{\sum_{i=1}^{N}{\left({\mathrm{DN}}_{i}-\bar{\mathrm{DN}}\right)}^{2}}{N}}$$

Land surface albedo is an important indicator that determines energy budget and the change of micrometeorological conditions like temperature, aridity/humidity etc. of land affected by desertification [13,32]. Some studies showed the increasing of land surface albedo implies a degradation of land quality [33]. In this study, we adopted broad-band albedo that determined by the combination of narrow-band albedo to assess the micrometeorological conditions of land surface. Narrow-band albedo for each band (except band 6 for Landsat TM/ETM+) of Landsat images was determined by using the dark object subtraction method technique [34,35]. Broad-band albedo was then calculated according to its relationship with each narrow-band [36,37]. The broad-band albedo calculation for Landsat MSS and Landsat TM/ETM+ are expressed by Formulas (3) and (4):

(3) $${\mathrm{Albedo}}_{\mathrm{MSS}}=0.228\alpha_{1}+0.217\alpha_{2}+0.182\alpha_{3}+0.373\alpha_{4}$$

(4) $${\mathrm{Albedo}}_{\mathrm{TM}/\mathrm{ETM}+}=0.356\alpha_{1}+0.130\alpha_{3}+0.373\alpha_{4}+0.085\alpha_{5}+0.072\alpha_{7}-0.0018$$

where *Albedo_MSS_* and *Albedo_TM / ETM+_* represent the broad-band albedo of Landsat MSS and Landsat TM/ETM+; *α*_1–7_ are narrow-band albedo for each band of Landsat MSS (band 1–4) and Landsat TM/ETM+.

### 3.3. Assessment Method

In this study, the land desertification of Ordos was classified into five grades: non-desertification, low desertification, medium desertification, high desertification and severe desertification according to a frequently-used grading system in China [38,39]. Our goal was to separate each desertification grade by using the differences among the indicators and its combinations of desertification land. Decision Tree (DT) is a suitable approach for classification because it can employ tree structured rules to recursively partition the data set feature spaces into increasingly homogenous classes based on a splitting criterion. DT classification has been extensively used for vegetation mapping, ecological modeling, soil mapping, and in remote sensing studies [40,41]. In this study, we used DT classification to assess the status of desertification of Ordos.

Information from ancillary data sources may help to increase assessment accuracy, especially in large area land-cover mappings that involve cover types characterized by high levels of within-class variability [42]. Land surface vegetation conditions in Ordos have great heterogeneity due to the rainfall and temperature gradients in this region [30], which may produce major classification errors when using the same rules for the whole region. Therefore, vegetation type was used as ancillary information for desertification assessment. Meanwhile, the date of image may also be served as ancillary data due to variations of indicators for a desertification grade at different times in a year.

According to the desertification classification system used in the National Key Basic Project “Process and Controlling of Aeolian Desertification in North China” [38,39], ground information has been used to define the training and accuracy checking sets, which would be used to make rule sets for assessment and accuracy checking. Fifty training sets and ten checking sets were selected for each grade of desertification in each vegetation sub-region (Figure 1) according to the date of the images. In order to represent more information, these sets must be equally distributed in a specific landscape. In this study, the temporal unit of images used to make rule sets was month and mainly between June and November. The surface information of desertification land used to define the training sets was extracted from the three indicator bands for all images. According to frequencies distribution of the five desertification grade for each vegetation sub-region in a certain period, the rules for each indicator can be established and the DT can be built. Images were classified in ERDAS by using the knowledge engineer module with total agreement of rules in assessment. The single assessed map of whole Ordos for each of the three periods was created by using mosaic images tool in ERDSA to merge all classified results of each period with selecting maximum function in overlap areas. Then accuracy was assessed and the training sets and rule sets might be adjusted according to visual examination and accuracy assessment until better assessment accuracy was achieved.

## 4. Results and Analysis

### 4.1. Desertification Assessment and Accuracy Checking

**Figure 2.**
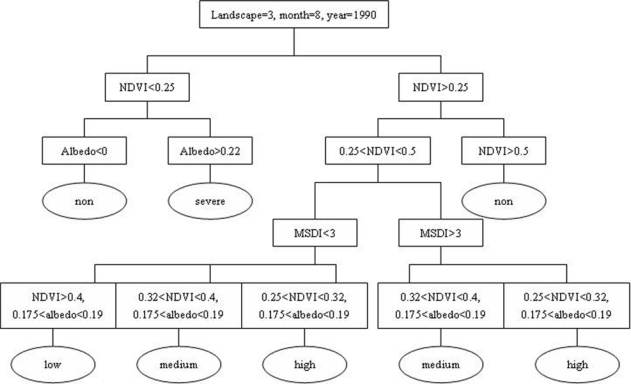
Decision Tree for desertification assessment of temperate deciduous scrubs sub-region (landscape = 3) in August, 1990. “non”, “low”, “medium”, “high” and “severe” are used to represent desertification grades for short. NDVI, MSDI and albedo are the indicators used to build the rules.

The rule sets of indicators for each grade of desertification in every vegetation sub-region are listed in Tables 1 and 2 (considering the huge size of the total rule sets, only rule sets for August, October and November for irrigated farmland, temperate deciduous scrubs and desert are listed). Great differences exist for defining a desertification grade from the three indictors at a regional scale due to various vegetation sub-regions, the date of images and different satellite sensors. According to the rule sets, the change of NDVI and albedo had a linear relationship with the change of desertification grade, which showed that the reversion of desertification was characterized by the increasing of NDVI or the decreasing of albedo. In this study, water body was classified into non-desertification and can be accurately classified by setting the rule of that region with negative value of NDVI. MSDI was used in this study to characterize desertification from the difference of landscape pattern or heterogeneity in different desertification grades. However, the change of landscape pattern or heterogeneity did not have a linear relationship with the change of desertification. The change of landscape pattern or heterogeneity in desertification land always had a trend like a parabola, which indicated that the MSDI of desertification land initially increased and then declined with the expansion of desertification. In this study, because severe and non-desertification can be accurately identified by using only NDVI and albedo, MSDI was mainly used to distinguish the low, medium and high desertification. The rule sets for different desertification grades varied greatly due to seasonal variation, especially for NDVI and albedo, which had a higher NDVI value and lower albedo value in summer (August) than that in autumn (October and November) respectively. According to the rule sets, Decision Tree was built to assess the status of desertification of Ordos. The Decision Tree model for desertification assessment in temperate deciduous scrubs sub-region in August by using Landsat TM/ETM+ was shown in Figure 2, where landscape 3 is the temperate deciduous scrubs sub-region. Models for desertification assessment in other sub-regions were developed in a similar manner as in Figure 2 and are not presented here.

Accuracy checking of assessing results for 1980, 1990 and 2000 had been carried out by using confusion matrix. A confusion matrix for accuracy checking of desertification assessment results in the three periods was listed in Table 3. The satisfactory classification results have been obtained with the average overall accuracy of three periods higher than 0.9 and the highest overall accuracy and kappa statistic in the assessment for 1990, which reached 0.926 and 0.9075 respectively. Due to the relative low quality of images in 2000, the overall accuracy and kappa statistic were the lowest, which reached 0.9 and 0.875 respectively. The overall accuracy and kappa statistic in the assessment for 1980 were little higher than that of 2000. For the accuracy of each grade, the severe desertification grade was total correctly assessed and this might attribute to that severe desertification land covered by drifting sand dunes, which was characterized by the lowest NDVI and highest albedo that made these regions easily and accurately classified. The land with a medium desertification grade always had the most complex land surface condition, which made some parts of these regions classified into low and high desertification grade and the accuracy of medium desertification having the lowest, albeit still good, accuracy.

### 4.2. The Processes and Causes of Desertification from 1980 to 2000

**Figure 3.**
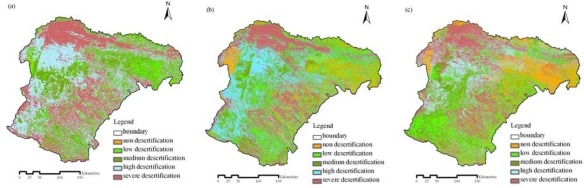
Desertification maps of Ordos in 1980 (a), 1990 (b) and 2000 (c).

**Figure 4.**
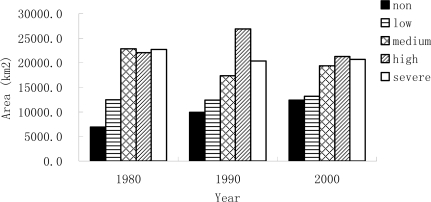
Statistics of the desertification area of Ordos in 1980, 1990 and 2000. “non”, “low”, “medium”, “high” and “severe” are used to represent desertification grades for short.

The assessment results of desertification in 1980, 1990 and 2000 are shown in Figure 3. Owing to the differences of spatial resolution between Landsat MSS (57 m) and TM/ETM+ (28.5 m), the assessment result of desertification in 1980 was resampled to the same resolution as that in 1990 and 2000 for comparison. According to areas of different desertification grades in three periods (Figure 4), the desertification of Ordos displayed a reversing trend, although some local places experienced a desertification expansion from 1980 to 2000. From 1980 to 2000, the area of non-desertification had a significant increase and low desertification had changed little. Based on analyzing the area and percentage of regions experienced desertification reversion and expansion in each sub-region (Table 4), all sub-regions had a reversing trend except steppe shrub sub-region from 1980 to 1990 and the proportion of reversing desertification in irrigated farmland was higher than that of other sub-regions. From 1990 to 2000, desertification in irrigated farmland and desert sub-regions had an expanding trend; however the other three sub-regions experienced a reversing trend with the highest reversing percentage found in the temperate steppes.

**Figure 5.**
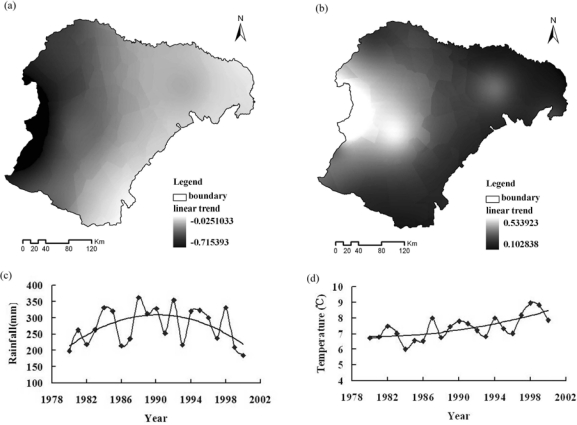
The linear trend of aridity/humidity index in Ordos from 1980 to 1990 (a), from 1990 to 2000 (b); the change of rainfall (c) and temperature (d) in Ordos from 1980 to 2000.

The change of desertification is caused by both climate change and human activities. In this study, climate data from thirty-four meteorological stations in and around Ordos and some social-economic indicator were used to analyze the impact of climate change and human activities on desertification from 1980 to 2000. Aridity/humidity conditions of land surface can reflect the integrated impact of various climate factors [43], which can be used to explain the effect of climate change on vegetation growth and the processes of desertification. To investigate the impacts of climate change on desertification, aridity/humidity index that calculated as the ratio of potential evapotranspiration to rainfall [44] was used to reflect the effects of climate change on aridity/humidity condition of land surface and the processes of desertification. In this study, climate data from thirty-four meteorological stations in and around Ordos were interpolated to grid data with 1 km resolution and a linear trend of aridity/humidity index in every pixel was calculated to analyze the impacts of climate change in Ordos from a spatial point of view [Figures 5(a) and (b)].

**Figure 6.**
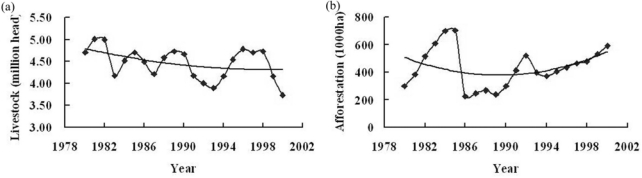
The change of livestock number **(**a) and the area of afforestation (b) in Ordos from 1980 to 2000.

Meanwhile, the trend of regional averaged rainfall and temperature were also illustrated [Figures 5(c) and (d)]. Due to the social-economic data are always collected based on administrative unit, so the sum of social-economic indicator data (livestock number and area of afforestation) of all counties in Ordos was used in temporal serial analysis to reflect the impacts of human activities on desertification (Figure 6). From 1980 to 1990, although the temperature had increased somewhat, the rainfall had a significant increase and the aridity/humidity index of the whole region showed a decreasing trend, which indicated the climate of this period was more humid and would benefit vegetation growth and the reversion of desertification. The change of human activities in this period showed that although livestock number decreased somewhat, the area of afforestation decreased significant especially in the late of 1980s; meanwhile, related vegetation protection policies were not extensively conducted in this period and land-use was still intensive. Based on integrated analysis of the trend and characteristics of climate change and human activities, human activities might explain the expansion of desertification in Ordos from 1980 to 1990 at a regional scale. From 1990 to 2000, the temperature showed an increasing trend and the rainfall had a decreasing trend, which made the aridity/humidity index of the whole region show an increasing trend and it indicated the climate of this period was more arid and would speed up the expansion of desertification. Meanwhile, livestock numbers in Ordos decreased gradually and the area of afforestation had an obviously increase in this period, which might attribute to ecological projects such as Three-North Forest Shelterbelt Program conducted in Ordos since 1990s [45,46]. Based on integrated analysis of the trend and characteristics of climate change and human activities, human activities might explain the reversion of desertification in Ordos from 1980 to1990 at a regional scale.

## 5. Conclusions

This paper focused on assessing desertification from a quantitative viewpoint by means of remote sensing. In this study, assessing indicators were selected according to three different aspects from the responses of land surface conditions to desertification. Based on considering the differences of indicators for assessing desertification in different region and time, vegetation or landscape types, and dates of image acquisition were employed for building rule sets in a Decision Tree model at a regional scale, which improved the accuracy of assessment. In this study, average overall accuracy of assessments in three periods was higher than 90%.

Although some local places of Ordos experienced an expansion of desertification, the trend of desertification of Ordos was an overall decrease in from 1980 to 2000. For the change of desertification in sub-regions, temperate steppe and temperate deciduous scrubs sub-regions showed a stable reversion of desertification from 1980 to 2000; however, the other sub-regions did not show a stable reversion trend in this 20-year period. The reversing trend of desertification from 1980 to 1990 was mainly induced by climate change; however human activity was the factor that dominated the reversion of desertification from 1990 to 2000 at a regional scale.

The concepts and methods put forward in this study not only can quantitatively assess desertification at a regional scale, but also can be used in other land degradation assessment at different temporal and spatial scales. Improvement of the indicator system and use of more ancillary data may be helpful to improve the assessing accuracy.

## Figures and Tables

**Table 1.** The rule sets for desertification assessment by using Landsat MSS images.

**Month**	**Desertification Grade**	**Irrigated farmland**			**Temperate deciduous scrubs**			**Desert**		

		NDVI	MSDI	Albedo	NDVI	MSDI	Albedo	NDVI	MSDI	Albedo

August	non	<0 or >0.45	>1	0–0.22	<0 or >0.45	>1	0–0.19	<0.2 or >0.4	>2	0–0.24
	low	0.35–0.45	0–4	0.22–0.24	0.3–0.45	0–4	0.19–0.21	0.28–0.4	0–8	0.24–0.26
	medium	0.23–0.35	0–6	0.24–0.26	0.2–0.3	0–7	0.21–0.23	0.18–0.28	0–6	0.26–0.28
	high	0.1–0.23	0–5	0.26–0.28	0.1–0.2	0–5	0.23–0.26	0.06–0.18	0–4	0.28–0.30
	severe	0–0.1	0–3	>0.28	0–0.1	>1	>0.26	0–0.06	0–3	>0.30
October	non	<0 or >0.3	>1	0–0.26	<0 or >0.35	>1	0–0.24	<0.15 or >0.3	>1	0–0.26
	low	0.2–0.3	0–5	0.26–0.29	0.22–0.35	0–3	0.24–0.27	0.2–0.3	0–5	0.26–0.29
	medium	0.145–0.20	0–8	0.29–0.33	0.16–0.22	0–5	0.27–0.3	0.14–0.2	0–4	0.29–0.31
	high	0.085–0.145	0–6	0.33–0.36	0.08–0.16	0–4	0.3–0.33	0.04–0.14	0–3	0.31–0.34
	severe	0–0.085	0–4	>0.36	0–0.08	>1	>0.33	0–0.04	0–2	>0.34

“non”, “low”, “medium”, “high” and “severe” are used to represent desertification grades for short

**Table 2.** The rule sets for desertification assessment by using Landsat TM/ETM+ images.

**Month**	**Desertification Grade**	**Irrigated farmland**			**Temperate deciduous scrubs**			**Desert**		

		NDVI	MSDI	Albedo	NDVI	MSDI	Albedo	NDVI	MSDI	Albedo

August	non	<0 or >0.5	>1	0–0.16	<0 or >0.5	>1	0–0.175	<0.28 or >0.4	>1	0–0.2
	low	0.4–0.5	0–3	0.16–0.18	0.4–0.5	0–3	0.175–0.19	0.32–0.4	0–5	0.16–0.18
	medium	0.32–0.4	0–6	0.18–0.20	0.32–0.4	0–5	0.19–0.205	0.26–0.32	0–4	0.18–0.20
	high	0.24–0.32	0–4	0.20–0.22	0.25–0.32	0–4	0.205–0.22	0.22–0.26	0–3	0.20–0.22
	severe	0–0.24	0–3	>0.22	0–0.25	>1	>0.22	0–0.22	0–2	>0.22
November	non	<0 or >0.27	>1	0–0.35	<0 or >0.25	>1	0–0.35	<0.18 or >0.24	>3	0–0.37
	low	0.22–0.27	0–8	0.35–0.4	0.22–0.25	0–8	0.35–0.4	0.20–0.24	0–8	0.37–0.42
	medium	0.18–0.22	0–6	0.4–0.45	0.19–0.22	0–6	0.4–0.45	0.17–0.20	0–6	0.42–0.48
	high	0.15–0.18	0–5	0.45–0.5	0.16–0.19	0–4	0.45–0.5	0.15–0.17	0–5	0.48–0.52
	severe	0–0.15	0–4	>0.5	0–0.16	>1	>0.5	0–0.15	0–4	>0.52

“non”, “low”, “medium”, “high” and “severe” are used to represent desertification grades for short

**Table 3.** The confusion matrix and accuracy assessment for desertification in 1980, 1990 and 2000.

**Year**	**Desertification Grade**	**non**	**low**	**medium**	**high**	**severe**	**total**		**Producers accuracy**	**Users accuracy**
1980	non	94	4	2	0	0	100		94.00%	97.92%
	low	4	88	5	3	0	100		88.00%	88.00%
	medium	0	8	86	4	0	100		86.00%	85.15%
	high	0	4	6	87	3	100		87.00%	88.78%
	severe	0	0	0	0	100	100		100.00%	95.24%
	total	96	100	101	98	105	500			

Overall accuracy: 0.91; kappa statistic: 0.8875										

1990		non	low	medium	high	severe	total			
	non	94	5	1	0	0	100		94.00%	97.92%
	low	2	90	8	0	0	100		90.00%	90.00%
	medium	0	5	88	7	0	100		88.00%	87.13%
	high	0	0	4	91	5	100		91.00%	92.86%
	severe	0	0	0	0	100	100		100.00%	95.24%
	total	96	100	101	98	105	500			

Overall accuracy: 0.926; kappa statistic: 0.9075										

2000		non	low	medium	high	severe	total			
	non	92	3	4	1	0	100	92.00%		95.83%
	low	2	86	6	6	0	100	86.00%		86.00%
	medium	0	7	85	8	0	100	85.00%		84.16%
	high	0	3	7	87	3	100	87.00%		88.78%
	severe	0	0	0	0	100	100	100.00%		95.24%
	total	96	100	101	98	105	500			

Overall accuracy: 0.9; kappa statistic: 0.875										

Average overall accuracy of three periods: 91.2%; average kappa statistic of three periods: 0.89; “non”, “low”, “medium”, “high” and “severe” are used to represent desertification grades for short

**Table 4.** Area and percentage of desertification reversion and expansion in sub-regions from 1980 to 2000.

**sub-region**	**desertification change from 1980 to 1990**				**desertification change from 1990 to 2000**			

	reversed		expanded		reversed		expanded	

	Area (km^2^)	Percentage (%)	Area (km^2^)	Percentage (%)	Area (km^2^)	Percentage (%)	Area (km^2^)	Percentage (%)

irrigated farmland	1555.3	37.8%	1100.1	26.8%	1354.2	32.9%	1359.7	33.1%
temperate steppe	3240.2	34.6%	3091.1	33.0%	3708.5	39.6%	2604.9	27.8%
temperate deciduous scrubs	15601.5	28.0%	14594.1	26.2%	20057.5	36.0%	13132.3	23.6%
steppe shrub	1887.9	20.6%	2667.2	29.1%	3066.5	33.4%	2547.3	27.8%
desert	2037.7	23.7%	1816.0	21.2%	1965.8	22.9%	2086.9	24.3%
total	2432.6		23268.5		30152.5		21731.1	
